# Differential Patterns of Synaptotagmin7 mRNA Expression in Rats with Kainate- and Pilocarpine-Induced Seizures

**DOI:** 10.1371/journal.pone.0036114

**Published:** 2012-05-02

**Authors:** Gordana Glavan, Ronald Eugene See, Marko Živin

**Affiliations:** 1 Brain Research Laboratory, Institute of Pathophysiology, Medical Faculty, University of Ljubljana, Ljubljana, Slovenia; 2 Department of Neurosciences, Medical University of South Carolina, Charleston, South Carolina, United States of America; Karolinska Institute, Sweden

## Abstract

Previous studies in rat models of neurodegenerative disorders have shown disregulation of striatal synaptotagmin7 mRNA. Here we explored the expression of synaptotagmin7 mRNA in the brains of rats with seizures triggered by the glutamatergic agonist kainate (10 mg/kg) or by the muscarinic agonist pilocarpine (30 mg/kg) in LiCl (3 mEq/kg) pre-treated (24 h) rats, in a time-course experiment (30 min - 1 day). After kainate-induced seizures, synaptotagmin7 mRNA levels were transiently and uniformly increased throughout the dorsal and ventral striatum (accumbens) at 8 and 12 h, but not at 24 h, followed at 24 h by somewhat variable upregulation within different parts of the cerebral cortex, amigdala and thalamic nuclei, the hippocampus and the lateral septum. By contrast, after LiCl/pilocarpine-induced seizures, there was a more prolonged increase of striatal Synaptotagmin7 mRNA levels (at 8, 12 and 24 h), but only in the ventromedial striatum, while in some other of the aforementioned brain regions there was a decline to below the basal levels. After systemic post-treatment with muscarinic antagonist scopolamine in a dose of 2 mg/kg the seizures were either extinguished or attenuated. In scopolamine post-treated animals with extinguished seizures the striatal synaptotagmin7 mRNA levels (at 12 h after the onset of seizures) were not different from the levels in control animals without seizures, while in rats with attenuated seizures, the upregulation closely resembled kainate seizures-like pattern of striatal upregulation. In the dose of 1 mg/kg, scopolamine did not significantly affect the progression of pilocarpine-induced seizures or pilocarpine seizures-like pattern of striatal upregulation of synaptotagmin7 mRNA. In control experiments, equivalent doses of scopolamine *per se* did not affect the expression of synaptotagmin7 mRNA. We conclude that here described differential time course and pattern of synaptotagmin7 mRNA expression imply regional differences of pathophysiological brain activation and plasticity in these two models of seizures.

## Introduction

Synaptotagmin7 (Syt 7) is a member of the Syt family, which are proteins characterized as essential for synaptic and extrasynaptic membrane trafficking in the brain [1]–[3]. Syt 7 has been suggested to function as a regulator of synaptic vesicle exocytosis by working cooperatively with vesicular Syt 1 and Syt 2, based on its location on neuronal plasma membranes [4], [5]. Syt 7 protein has also been localized on lysosomes in primary sympathetic neurons [6]. The involvement of Syt 7 in exocytosis of lysosomes was confirmed in Syt 7-deficient mice that exhibited impaired membrane resealing, autoimmune myositis, and impaired insulin secretion and glucose intolerance [7], [8]. Superior cervical ganglion neurons explanted from these mice revealed marked defects in neurite outgrowth and arborization, suggesting that Ca^2+^-dependent, Syt 7-regulated exocytosis of late endosomes/lysosomes plays a role in the addition of new membrane for developing neurite extensions [6].

We previously showed dopamine dependent plasticity of Syt 7 expression in hypersensitive striatum in animal models of Parkinson's disease [9]–[11]. Specifically, antiparkinsonian drugs induced a robust increase in striatal Syt 7 mRNA levels in 6-OHDA and reserpinized rats, two established models for Parkinson's disease, *via* a dopamine D1 receptor-linked mechanism [9], [10]. These results led to the hypothesis that Syt 7 may play a significant role in the effectiveness of antiparkinsonian drugs [9], [10]. However, the exact molecular and pharmacological mechanisms by which changes in striatal Syt 7 expression may be involved in the dopaminergically deafferented striatum remain largely unknown.

Given the potential importance of Syt 7 regulation in pathological processes of brain plasticity, it would be expected that Syt 7 may also play a key role in epileptogenesis. Systemic administration of the convulsants, kainate (KA) or pilocarpine (PI) causes neuronal hyperexcitation and sustained seizure activity, which leads to excitotoxic injury, neurodegeneration, aberrant hippocampal plasticity, and gliosis [12]–[14]. These changes are followed by the development of spontaneous recurrent seizures, reminiscent of temporal lobe epilepsy in humans [15]. Although PI and KA both cause similar epileptic seizures and pathological changes in the brain, they differ in their initial mode of action. PI is a selective agonist of muscarinic acetylcholine receptors (mAChR) that provokes seizures indirectly by overstimulation of glutamatergic neurons of the cortex [16]. Once triggered by the excessive cholinergic activity, seizures are propagated by the sustained cortical glutamatergic overactivity. In the PI model, LiCl pre-treatment is given in order to decrease the threshold for the onset of seizures triggered by PI. In contrast, KA is agonist of some subtypes of glutamate receptors (GluR), and thus directly overstimulates the cortex by mimicking cortical glutamatergic overactivity [17].

In order to study the function of Syt 7 in seizures, we investigated whether epileptic seizures induced with either LiCl/PI or KA would affect the levels of Syt 7 mRNAs in the rat brain. In this study, we found that PI and KA induced differential patterns of upregulation of striatal Syt 7 mRNA. Furthermore, in rats with PI-induced seizures, the muscarinic antagonist, scopolamine (SCO), modified the pattern of striatal Syt 7 mRNA expression.

## Results

### Changes in Syt 7 mRNA levels in animals with KA-induced seizures

**Figure 1:**
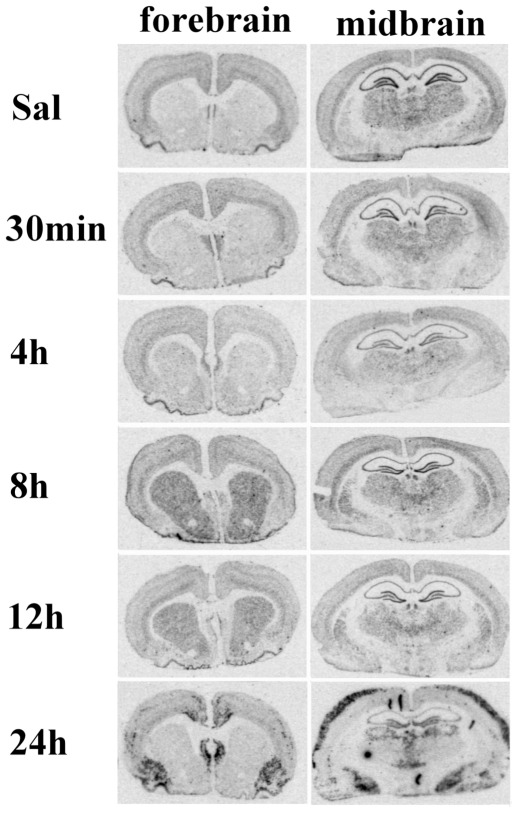
Time-course of Syt 7 mRNA expression in midbrain and forebrain after kainic acid-induced seizures or saline treatment. Animals were sacrificed at 30 min, 4, 8, 12, and 24 h after the injection.

**Figure 2:**
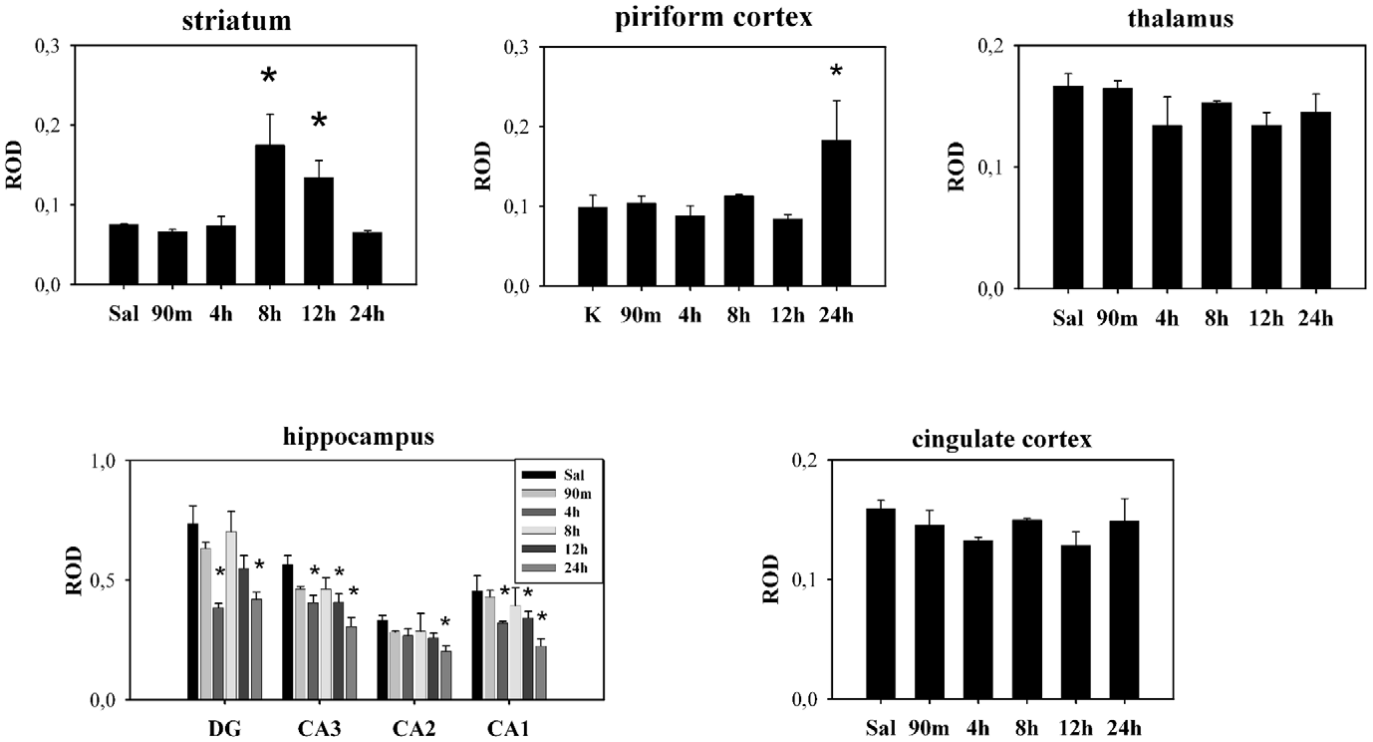
Levels of Syt 7 mRNA expression in different brain regions after kainic acid-induced seizures. ROD (relative optical density, means ± SEM) *significantly different from saline treated animals (one-way ANOVA followed by Tukey multiple-comparison test, *P*<0.05).

We examined a detailed time-course of the Syt7 mRNA signal for groups of rats that were killed 30 min, 4 h, 8 h, 12 h, and 1 day after the beginning of KA-evoked grade IV–V seizures [18]. In the dorsal striatum and ventral striatum (nucleus accumbens), we found a significant augmentation (one-way ANOVA followed by Tukey multiple-comparison test, *P*<0.05) of Syt 7 mRNA signal at 8 h and 12 h after the onset of KA-induced seizures as compared to controls (Figs. 1 and 2). In addition, KA induced a distinctive pattern of elevation of Syt 7 mRNA signal in several extrastriatal brain regions at 24 h *post* injection (Fig. 1). At 24 h, somewhat variable pattern of significant upregulation (one-way ANOVA followed by Tukey multiple-comparison test, *P*<0.05) was evident in the outer layers of the cerebral cortex, pyriform cortex, amigdala nuclei and the septum, while in the cingular cortex and thalamic nuclei the upregulation was not observed in all of the animals and it did not reach statistical significance (Figs. 1 and 2). In the rostral hippocampus, we observed a progressive significant decline (one-way ANOVA followed by Tukey multiple-comparison test, *P*<0.05) of Syt 7 mRNA signal t hat was most prominent at later time points (Figs. 1 and 2). However, in the dorsal hippocampus, a patchy upregulation pattern was observed in CA1, CA2 and CA3 regions of some of the animals, but again only at 24 h after the onset of grade IV–V seizures (not shown).

### Changes in Syt 7 mRNA levels in animals with LiCl/PI-induced seizures

**Figure 3:**
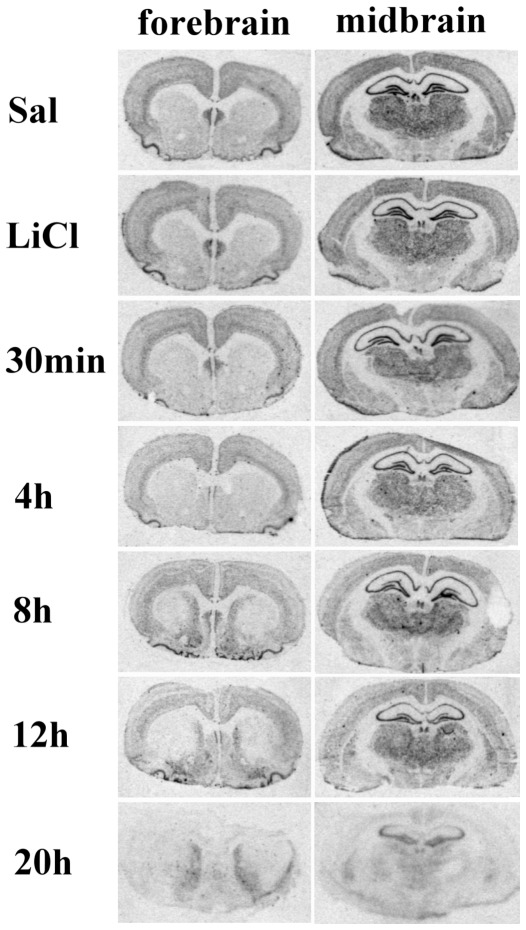
Time-course of Syt 7 mRNA expression in midbrain and forebrain after pilocarpine-induced seizures. Animals were killed 30 min, 4, 8, 12, and 20 h after the injection, and two groups of animals received saline injection (Sal) or LiCl.

**Figure 4:**
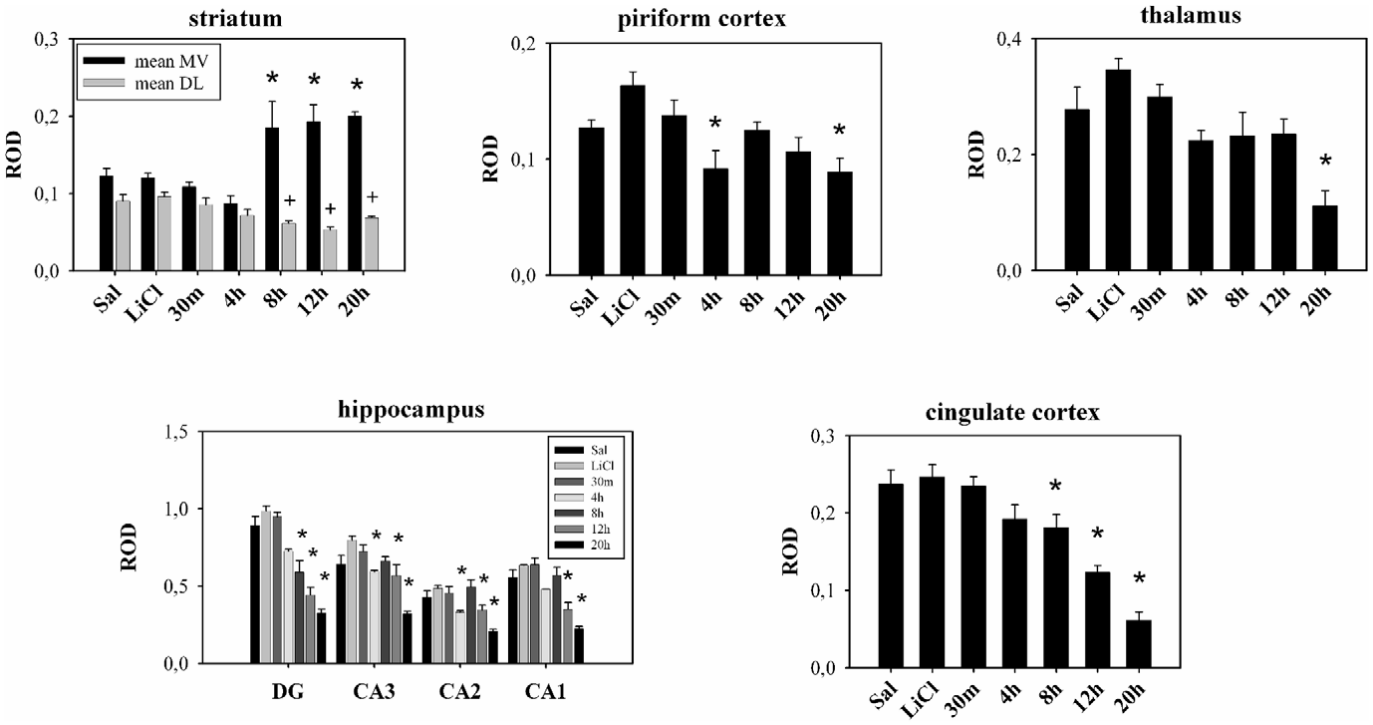
Levels of Syt 7 mRNA expression in different brain regions after pilocarpine-induced seizures. VM-ventromedial striatum; DL-dorsolateral striatum. ROD (relative optical density, means ± SEM) *significantly different from the same brain region of saline treated animals (one-way ANOVA followed by Tukey multiple-comparison test, *P*<0.05). ^+^significantly different from the dorsolateral striatum of saline treated animals (one-way ANOVA followed by Tukey multiple-comparison test, *P*<0.05).

In the time-course experiment for LiCl/PI-induced seizures, Syt 7 mRNA levels were determined at 30 min, 4 h, 8 h, 12 h, and approximately 1 day (20 h) after the onset of grade IV–V seizures. In the ventromedial striatum, LiCl/PI treatment induced a significant augmentation (one-way ANOVA followed by Tukey multiple-comparison test, *P*<0.05) of Syt 7 mRNA signal at 8, 12, and 20 h groups as compared to the control group (Figs. 3 and 4). In contrast, in the dorsolateral striatum, LiCl/PI significantly downregulated (one-way ANOVA followed by Tukey multiple-comparison test, *P*<0.05) the Syt 7 mRNA signal at 8, 12 and 20 h as compared to the control group (Figs. 3 and 4). Significant downregulation (one-way ANOVA followed by Tukey multiple-comparison test, *P*<0.05) of Syt 7 mRNA levels was also observed at later time points in other brain regions: piriform and cingulate cortex, thalamus and dentate gyrus, CA3, CA2, CA1 regions of hippocampus (Fig. 4). There were no significant differences (paired Student's t-test, p>0.05) of corresponding Syt 7 mRNA signals between two control groups, saline-only and LiCl-only treated animals, in any of the observed brain regions (Fig. 4).

### Effect of scopolamine post-treatment on Syt 7 mRNA levels in animals with seizures induced with LiCl/PI

**Figure 5:**
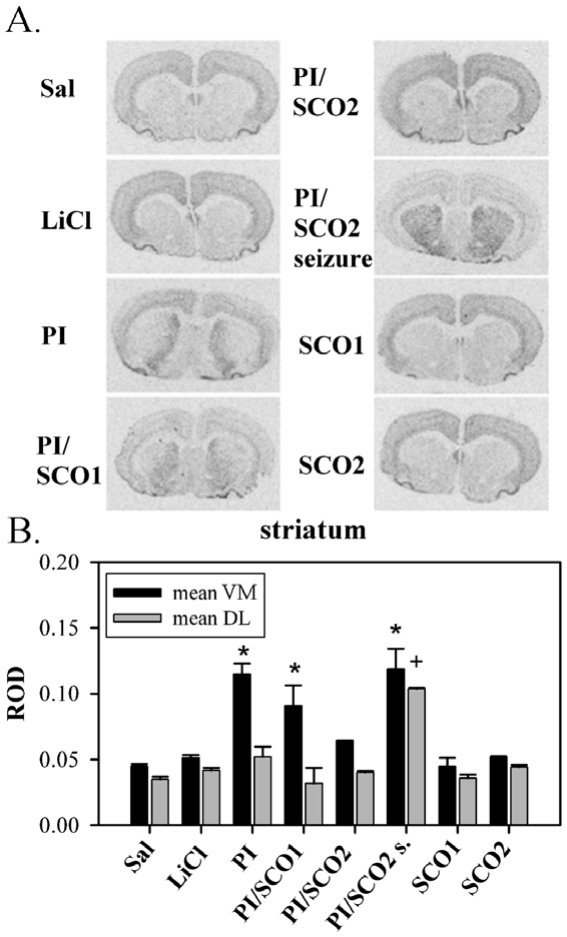
Effect of scopolamine on striatal Syt 7 mRNA expression after pilocarpine treatment. Two groups of animals received injection of 1 mg/kg (PI/SCO1) or 2 mg/kg (PI/SCO2) of scopolamine after displaying the first signs of pilocarpine-induced seizures. Epileptic seizures were either prevented (PI/SCO2) or were not prevented (PI/SCO2 seizure) by scopolamine (2 mg/kg). Another three groups received either saline (Sal), LiCl, or pilocarpine together with LiCl (PI). All animals were killed 12 h after the treatment. A) Representative autoradiograms showing striatal Syt 7 mRNA expression after different treatments. B) ROD measurements (means ± SEM) showing striatal levels of Syt 7 mRNA expression. VM-ventromedial striatum; DL-dorsolateral striatum. *significantly different that ROD measured in the same brain region of saline treated animals (one-way ANOVA followed by Tukey multiple-comparison test, *P*<0.05). ^+^significantly different from ROD measured in dorsolateral striatum of saline treated animals (one-way ANOVA followed by Tukey multiple-comparison test, *P*<0.05).

The treatment with 2 mg/kg, but not with 1 mg/kg SCO abolished the LiCl/PI induced grade IV seizures in two out of four animals. In these animals the Syt 7 mRNA expression pattern (observed 12 h after the onset of seizures) was similar to controls (Fig. 5; one-way ANOVA followed by Tukey multiple-comparison test, *P*<0.05). In the remaining two animals from this group further seizure activity was attenuated by post-treatment with 2 mg/kg SCO, Syt 7 mRNA was upregulated throughout the dorsal and ventral striatum in a manner similar to that in KA group (Fig. 5). By contrast, in animals post-treated with 1 mg/kg of SCO at the onset of LiCl/PI-induced seizures, the seizures continued unabated during the 1 h observation period with apparently unchanged intensity. In these animals Syt 7 mRNA was upregulated only in the ventromedial striatum (as observed 12 h after the onset of seizures) in a manner similar to that observed in the LiCl/PI group of animals with seizures without scopolamine post-treatment (Fig. 5). There were no significant differences (one-way ANOVA followed by Tukey multiple-comparison test, *P*<0.05) of corresponding Syt 7 mRNA ROD signals between different control groups (*i.e.* animals treated with only saline, LiCl, scopolamine 1 mg/kg or 2 mg/kg) (Fig. 5).

## Discussion

In the present study, we used *in situ* hybridization with a Syt 7 gene specific probe to investigate whether epileptic seizures induced by PI or KA would affect the levels of Syt 7 mRNA in the brain and to investigate some of the possible pharmacological mechanisms underlying the seizure-induced changes of striatal Syt 7 mRNA expression. To the best of our knowledge this study demonstrates for the first time that brain Syt 7 mRNA levels could be strongly affected in rat models of seizures. After KA-induced seizures, Syt 7 mRNA levels were transiently and uniformly increased throughout the striatum, followed by somewhat variable upregulation within different parts of the cerebral cortex, amigdala and thalamic nuclei, the caudal (but not rostral) hippocampus and the lateral septum. By contrast, after PI-induced seizures, there was a more prolonged increase of Syt 7 mRNA levels, but only in the ventromedial striatum, while in the dorsolateral striatum and in some other of the above mentioned brain regions there was concomittant downregulation of Syt 7 mRNA levels to below the basal levels.

In both seizure models, the striatum stood out as the region where the changes in Syt 7 mRNA expression appeared first. It is well known that the striatum regulates motor coordination and is connected with cortical and subcortical structures that are activated during seizures [19], [20]. The striato–nigral GABAergic neurons may be important in the seizure activity formation, since the activation of these neurons by NMDA agonists or GABA antagonists block seizures [16], [21]. The striatum might serve as as relay or gating station for the propagation of seizures [22], a role consistent with the known functional connections of the basal ganglia with thalamo-cortical and the limbic system. In our experiments, KA-induced seizures upregulated Syt 7 mRNA uniformly across the striatum and nucleus accumbens, whereas PI-evoked seizures upregulated Syt 7 mRNA only within the ventromedial striatum. Moreover, we found a downregulation of Syt 7 mRNA signal in the dorsolateral striatum at 12 h after the beginning of PI-induced seizures. The reason for these differences between the two models likely relates to the different receptor mechanisms by which PI and KA induce seizures. KA directly stimulates kainate and a′-amino-3-hydroxy-5-methylisoxazole-4-propionic acid (AMPA) receptors, whereas PI causes neuronal hyperexcitation *via* muscarinic receptor activation. All three types of receptors have been localized in the striatum, including all five muscarinic subtypes, M1-M5 [23]–[25]. Striatal AMPA, kainate, and muscarinic receptors are expressed on intrinsic postsynaptic neurons [24], which contain soma in the striatum. These neurons comprise mainly GABAergic medium-spiny neurons that project to substantia nigra reticulata and globus pallidus, as well as cholinergic interneurons. Thus, PI and KA, at least to a certain extent, may induce Syt 7 mRNA expression in the striatum by directly stimulating these receptors. As evidenced by many studies, KA causes the upregulation of numerous genes in hippocampus directly by stimulation of glutamate receptors [26]. We have also found the upregulation of Syt 7 mRNA after KA-induced seizure in some of epileptogenic regions normally affected by glutamate transmission, such as the caudal hippocampus, amigdala, pyriform cortex and the thalamus. However, our results do not exclude the possibility that Syt 7 mRNA expression may be regulated by mechanisms other than by direct stimulation of glutamate and/or muscarinic receptors. For example, the upregulation of Syt 7 mRNA in KA model of seizures that occurred in many regions with strong dopaminergic innervations, such as striatum and cingular cortex, may be tentatively explained also by dopaminergic hyperactivity during seizures. Indeed, it has been reported that induction of seizure activity by both KA and PI can affect gene expression in striatal neurons indirectly by increasing striatal dopamine release [27]–[29]. PI induces striatal Fos protein expression probably indirectly *via* D1 receptor activation, since it can be almost completely prevented by of the D1 antagonist, SCH-23390 [30]. Striatal D1 receptor activation causes the upregulation of Fos and Jun proteins which bind AP-1 transcription complex-binding consensus sequences in promoter areas of several genes [31], [32]. D1 receptor stimulation also causes the phosphorylation of other transcription factors, such as cAMP response element-binding protein (CREB). Interestingly, an important role of the CREB/CRE transcriptional pathway has been recently suggested to mediate the expression of genes responsible for the protection against oxidative stress-mediated neuronal cell death in the PI model [33]. A search of the GeneBank database indicates that the Syt 7 gene actually possesses both the cAMP-response element binding protein (CRE) and AP-1 transcription complex-binding consensus sequences in its promoter area, which bind CREB and Fos/Jun proteins. Syt 7 expression in striatum has previously been shown to be regulated by dopamine *via* D1, but not D2, receptors [9], [10]. Namely, in dopamine deprived striatum of hemiparkinsonic rats, striatal Syt 7 mRNA also attained peak levels between 8 and 12 h after D1 receptor stimulation [9]. This suggests that in PI- and KA induced seizures, Syt 7 mRNA upregulation in the striatum may be provoked by increased striatal dopamine release during seizures. Moreover, the possibility of dopaminergic involvement may be inferred also by considering the longstanding hypothesis of an antagonistic balance between dopamine and acetylcholine in normal striatal function [34]. For example, cholinergic transmission *via* interaction with muscarinic receptors, inhibits basal and D1 receptor-stimulated striatal gene expression and cAMP formation [35], [36]. Taking into consideration the possibility of the above mentioned dopaminergic-cholinergic interactions, our results suggest that the PI-could prevent the effect of excessive release of endogenous dopamine during seizures on Syt 7 mRNA upregulation or even induce downregulation of Syt 7 mRNA in the dorsolateral striatum.

To further explore the proposed cholinergic mechanism of seizure-induced changes of striatal Syt 7 mRNA expression, we performed additional experiments with the aim to inhibit the effect of PI on muscarinic receptors by SCO post-treatment. A high systemic dose of SCO (2 mg/kg), given at the onset of PI-induced seizures, completely prevented the upregulation of Syt 7 mRNA in ventromedial striatum and nucleus accumbens, but only in rats in which SCO also completely abolished PI-induced seizures. In these animals, the pattern of striatal Syt 7 mRNA levels was similar to the pattern in control rats without seizures. In contrast, in animals in which the same high dose of SCO did not inhibit the seizures, Syt 7 mRNA showed robust upregulation across the whole striatum (i.e., not limited to the ventromedial striatum and nucleus accumbens, as seen in animals with PI-induced seizures). It may be possible that in these animals, the propagation of seizures was sustained by glutamatergic mechanisms, while SCO prevented further stimulation of muscarinic receptors by PI. Interestingly, the striatal expression pattern in these animals was identical to that induced by KA-evoked seizures. This suggests that in the PI model of seizures, striatal Syt 7 mRNA upregulation may be opposed by the stimulation of muscarinic receptors. By this rationale, SCO given at the onset of seizures could attenuate the inhibiting effect of PI on seizure-evoked upregulation of syt 7 mRNA within the dorsolateral striatum. Why the same effect of SCO did not occur also in the ventromedial striatum, remains to be determined. Moreover, to better resolve this issue and to distinct between the possible direct or indirect involvement of the striatal muscarinic receptors on the expression of Syt 7 mRNA, intrastriatal stereotaxic injection of SCO (e.g. *via* previously implanted intrastriatal catheters) at the onset of PI- and KA- induced seizures, should be performed in the future.

Compared to controls, KA-induced seizures downregulated Syt 7 mRNA only in the rostral hippocampus, whereas PI diminished the Syt 7 mRNA expression in hippocampus, cingulate cortex and thalamus. In all of these brain regions, the greatest downregulation occurred at about one day after the onset of seizures, possibly indicating the loss of nerve cells or a general transcriptional attenuation during the process of epileptogenesis [12], [37].

A robust upregulation of numerous genes caused by seizure activity could be directly involved in neuronal plasticty associated with epileptogenesis [26], [38]–[40], while others are probably implicated in neurodegeneration and gliosis caused by a sustained seizure-related neuronal hyperexcitation [26]. In our experiment, massive upregulation of Syt 7 mRNA within epileptogenic regions occurred only after seizures induced with KA. Future studies should determine whether differential upregulation of Syt 7 mRNA confers some important differences in these two models of epileptogenesis. Based on current knowledge about Syt 7 function in the brain, altered Syt 7 expression could affect the Syt 1/Syt2 regulated synaptic vesicle exocytosis resulting in changed synaptic transmission [4], [5], or it could alter the exocytosis of late endosomes/lysosomes enabling the addition of new membrane to form new extensions [6]. Based on our findings, striatal plasticity could be differentially affected in these two models of seizures.

In conclusion, we found that seizures induced by KA and PI caused differential changes in the expression of Syt 7 mRNA in the brain, thus demonstrating complex regulatory mechanisms for striatal Syt 7 mRNA expression in seizures. It appears that the stimulation of muscarinic receptors with PI opposes, rather than stimulates the upregulation of striatal Syt 7 mRNA. We speculate that in both models of seizures, striatal upregulation of Syt 7 mRNA may be induced indirectly, possibly by the seizure-induced increases in striatal glutamatergic/dopaminergic transmission. The differential pattern of striatal Syt 7 mRNA upregulation may thus indicate cholinergic inhibition of striatal Syt 7 mRNA expression. Based on the possible role of Syt 7 in membrane reshaping and/or the regulation of synaptic transmission, this study also suggests differential plasticity within epileptogenic brain regions in kainate and pilocarpine models of seizures. This may differentialy affect the neuronal loss, reactive gliosis, and axonal sprouting that preceed the emergence of chronic spontaneous recurrent seizures [15] and implies at least subtle differences in these two models of human acquired epilepsy. Future progress in the understanding of Syt 7 regulation may thus shed new light on epileptogenesis and its potential treatment.

## Methods

### Animals

Adult male Wistar rats (290–330 g) were maintained on a 12 hr light/dark cycle (lights on 07:00–19:00) in a temperature-controlled colony room at 22–24°C with free access to rodent pellets and tap water. Subjects were handled according to the NIH Guide for the Care and Use of Laboratory Animals and all experiments were carried out in accordance with the European Council Directive of November 24th, 1986 (86/609/EEC).

### Drugs

The following drugs were used: lithium chloride (LiCl), pilocarpine hydrochloride (PI), scopolamine hydrobromide (SCO) and kainic acid; (KA; 2-Carboxy-3-carboxymethyl-4-isopropenylpyrrolidine hydrate), all purchased from Sigma, St. Louis, MO, USA. All drugs were dissolved in 0.9% saline (Sal) and were administered intraperitonealy (*i.p.*) in a volume of 1 ml/kg. Drug solutions were freshly prepared no more than 30 min before the injections.

### Behavioral observation

The behavior was recorded by a trained investigator that was not aware of the treatments. The rats were put in individual transparent cages (48 cm×20 cm×23 cm). By behavioral observation we aimed to select only the animals that developed Racine grade IV–V seizures [18]. We therefore ranked seizures as follows: − absent, + short bursts of convulsive activity (forelimb clonus with rearing, *i.e.* Racine grade IV), ++ status epilepticus (pronounced and prolonged convulsive activity, loss of balance and falling, *i.e.* Racine grade V).

### Time-course of Syt 7 mRNA expression in PI- and KA-induced epileptic seizures

In a time-course study, groups of four animals were sacrificed at different time points (30 min, 4 h, 8 h, 12 h and approximately 1 day (20–24 h) after the beginning of grade IV–V seizures according to the Racine scale induced by KA or LiCl/PI. In KA experiment, animals were treated with 10 mg/kg of kainic acid. The behavior during the “latent” period was similar, with few distinctive characteristics, depending on the seizure-inducing agent. For example, PI induced characteristic yawning-like oral dyskinesia, while KA in our hands induced more frequent wed-dog like shaking (not quantified). KA-induced grade IV seizures (rearing with forelimb clonus) with the onset in about 60 min. In control experiment a group four animals received control injection of Sal and were killed after 9 h (we took in the account the additional “latent” period of about 60 min before the start of grade IV–V seizure activity, as observed in the corresponding experimental 8 h group with KA-induced seizures). In the LiCl/PI experiment, animals were pretreated with LiCl (3 mEq/kg) 24 h before the injection of PI (30 mg/kg). Grade IV–V seizures started about 20 min after the injection of PI. Control animals in this experiment were pretreated with LiCl (3 mEq/kg) 24 h before the injection of Sal and were sacrificed 8 h and 20 min h after the injection of Sal (1 ml/kg) (we took in the account the additional “latent” period of about 20 min before the start of grade IV–V seizure activity, as observed in the corresponding experimental 8 h group with LiCl/PI-induced seizures). After injection of the seizure-inducing agent (or control injection of Sal) the animals were housed in individual cages and observed for 2 h.

### Scopolamine effects on Syt 7 mRNA expression in PI-induced seizures

For assessment of sistemicaly applied scopolamine post-treatment effects on Syt 7 mRNA expression in PI-induced seizures, animals were divided into seven groups. The first group (*n* = 4) received Sal injection (1 ml/kg) and served as the control group. The second group (*n* = 4) received LiCl (3 mEq/kg) 24 h before Sal (1 ml/kg) and also served as a control group. Animals from these control groups were killed 12 h after the injection of Sal. The third group (*n* = 4) was pretreated with LiCl (3 mEq/kg) 24 h before the injection of PI (30 mg/kg). All the animals in this group developed grade IV–V epileptic seizures approximately 20 min after the injection of PI and were sacrificed 12 h after the beginning of seizures. The fourth group (*n* = 4) was pretreated with LiCl (3 mEq/kg) 24 h before the injection of PI (30 mg/kg). When animals from this group started to display first signs of grade IV seizures, they received systemic injection of SCO (2 mg/kg). Group five (*n* = 4) received the same treatment as group four, except for receiving a lower dose of SCO (1 mg/kg)4. Group six (*n* = 3) received SCO (2 mg/kg) 24 h after the pre-treatment with LiCl (3 mEq/kg) and group seven (*n* = 3) received SCO (1 mg/kg) 24 h after the pre-treatment with LiCl (3 mEq/kg). Animals from groups four, five, six and seven were killed 12 h after the injection of SCO. After injection of the seizure-inducing agent (or control injection of SCO) the animals were housed in individual cages and monitored for 2 h for the presence or absence of grade IV–V seizures.

### Brain preparation and in situ hybridization histochemistry

The brains were removed and quickly frozen on dry ice. Coronal sections (10 µm) were cut through the neostriatum and hippocampus using a cryostat and thaw mounted onto microscope slides. The sections were then fixed in 4% phosphate-buffered paraformaldehyde, washed in phosphate-buffered saline, dehydrated in 70% ethanol, and stored in 95% ethanol at +4°C until used for *in situ* hybridization histochemistry as previously described in detail [41]. The sections were incubated with 3′ end ^35^S-labelled oligodeoxyribonucleotide antisense probes (45 bases long) complementary to the rat Syt 7 mRNA (bases encoding 300–344, sequence 5 = ′CCG AGT CTG GCG TGC CCA CCG TCT CCA AGG AGT TCT TGT AGC GTT-3′: the probe that recognizes all Syt 7 splicing variants), GenBank accession number U20106. Air dried sections were incubated with ^35^S-labeled probe in hybridization buffer containing 4× SSC (1× SSC is made out of 150 mM sodium chloride and 15 mM sodium citrate), 50% deionized formamide, 50 mM sodium phosphate (pH 7.0), 5× Denhardt's solution, 100 µg/ml polyadenylic acid, 10% dextran sulfate and 40 mM dithiothreitol. The oligodeoxynucleotide probes were labeled by incubation for one hour at 36°C with [35S]-deoxyadenosine 5′(-α-thio) triphosphate ([35S]-dATP; 1000–1500 Ci/mmol; DuPont NEN, Life Science Products Inc., Boston) and terminal deoxynucleotidyl transferase enzyme (Promega, Madison, WI, USA). The labeled probes were purified using spin columns with Sephadex G50. Specific activities of the labeled probes ranged from 55 to 150×10^3^ d.p.m./µl. Hybridization buffer with labeled probe was applied to each slide and incubated for 16 hr at 42°C in a humid chamber. Washing was performed for 30 min at room temperature followed by 1 hr wash at 55°C in 1× SSC. The sections were then quickly dipped in 0.1× SSC and dehydrated through 50%, 70%, and 98% ethanol. Air dried hybridized sections were exposed to X-ray film (Scientific Imaging Film X-Omat™ AR, Kodak, Rochester, NY) that were exposed for 2–3 weeks and developed using standard darkroom techniques. The specificity of the probe used in this study was confirmed by the almost complete disappearance of the autoradiographic signal when radiolabeled probe was hybridized in the presence of 100-fold excess of unlabeled probes (data not shown).

### Image analysis

The Syt 7 hybridization signal was analyzed using densitometry with MCID, M4 image analyzer (Imaging Research Inc., Canada) in the manually outlined regions of the ventromedial and dorsolateral striatum, thalamus, piriform and cingulate cortex, and hippocampal subregions (CA1, CA2, CA3 and dentate gyrus (DG)). Relative optical density (ROD) measurements in the striatum were performed on three sections of each animal. Nonspecific background signal, defined as the ROD of parts of the film without hybridization signal, was subtracted from the ROD measurements. *In situ* hybridization data was assessed with one-way analysis of variance (ANOVA) followed by Tukey multiple comparison test. In the experiments with PI, paired Student's t-test was performed to evaluate the difference by comparing ROD measurements of two control groups, saline and LiCl treated animals. All data are expressed as means ± S.E.M. with statistical significance set at *P*<0.05.

## References

[1] Südhof TC, Rizo J (1996). Synaptotagmins: C2-domain proteins that regulate membrane traffic.. Neuron.

[2] Marquèze B, Berton F, Seagar M (2000). Synaptotagmins in membrane traffic: which vesicles do the tagmins tag?. Biochimie.

[3] Fukuda M, Regazzi Romano (2006). The Role of Synaptotagmin and Synaptotagmin-Like Protein (Slp) in Regulated Exocytosis.. Molecular Mechanisms of Exocytosis.

[4] Sugita S, Han W, Butz S, Liu X, Fernández-Chacón R (2001). Synaptotagmin VII as a plasma membrane Ca(2+) sensor in exocytosis.. Neuron.

[5] Sugita S, Shin OH, Han W, Lao Y, Südhof TC (2002). Synaptotagmins form a hierarchy of exocytotic Ca2+ sensors with distinct Ca2+ affinities.. EMBO J.

[6] Arantes RM, Andrews NW (2006). A role for synaptotagmin VII-regulated exocytosis of lysosomes in neurite outgrowth from primary sympathetic neurons.. J Neurosci.

[7] Chakrabarti S, Kobayashi KS, Flavell RA, Marks CB, Miyake K (2003). Impaired membrane resealing and autoimmune myositis in synaptotagmin VII-deficient mice.. J Cell Biol.

[8] Gustavsson N, Lao Y, Maximov A, Chuang JC, Kostromina E (2008). Impaired insulin secretion and glucose intolerance in synaptotagmin-7 null mutant mice. Proc Natl Acad Sci.. U S A.

[9] Glavan G, Zivin M (2005). Differential expression of striatal synaptotagmin mRNA isoforms in hemiparkinsonian rats.. Neuroscience.

[10] Pal R, Zivin M, Milutinovic A, Jernej B, Glavan G (2007). Effect of apomorphine on striatal synaptotagmin 7 mRNA levels in reserpinized rats.. Neurosci Lett.

[11] Glavan G, Schliebs R, Zivin M (2009). Synaptotagmins in neurodegeneration.. Anat Rec (Hoboken).

[12] Clifford DB, Olney JW, Maniotis A, Collins RC, Zorumski CF (1987). The functional anatomy and pathology of lithium-pilocarpine and high-dose pilocarpine seizures.. Neuroscience.

[13] Pitkänen A, Schwartzkroin PA, Moshé SL (2006). Models of seizures and epilepsy.

[14] Danzer SC, He X, Loepke AW, McNamara JO (2010). Structural plasticity of dentate granule cell mossy fibers during the development of limbic epilepsy.. Hippocampus.

[15] Leite JP, Garcia-Cairasco N, Cavalheiro EA (2002). New insights from the use of pilocarpine and kainate models.. Epilepsy Res.

[16] Turski L, Cavalheiro EA, Schwarz M, Turski WA, De Moraes Mello LE (1986). Susceptibility to seizures produced by pilocarpine in rats after microinjection of isoniazid or gamma-vinyl-GABA into the substantia nigra.. Brain Res.

[17] Ben-Ari Y, Cossart R (2000). Kainate, a double agent that generates seizures: two decades of progress.. Trends Neurosci.

[18] Racine RJ (1972). Modification of seizure activity by electrical stimulation: II Motor seizures.. Electroencephalography and Clinical Neurophysiology.

[19] Kusske JA (1979). Corticocaudatothalamic interactions in experimental focal epilepsy in the cat.. Exp Neurol.

[20] Bonhaus DW, Walters JR, McNamara JO (1986). Activation of substantia nigra neurons: role in the propagation of seizures in kindled rats.. J Neurosci.

[21] Cavalheiro EA, Turski L (1986). Intrastriatal N-methyl-D-aspartate prevents amygdala kindled seizures in rats.. Brain Res.

[22] Slaght SJ, Paz T, Mahon S, Maurice N, Charpier S (2002). Functional organization of the circuits connecting the cerebral cortex and the basal ganglia: implications for the role of the basal ganglia in epilepsy.. Epileptic Disord.

[23] Levey AI, Kitt CA, Simonds WF, Price DL, Brann MR (1991). Identification and localization of muscarinic acetylcholine receptor proteins in brain with subtype-specific antibodies.. J Neurosci.

[24] Tarazi FI, Campbell A, Yeghiayan SK, Baldessarini RJ (1998). Localization of ionotropic glutamate receptors in caudate-putamen and nucleus accumbens septi of rat brain: comparison of NMDA, AMPA, and kainate receptors.. Synapse.

[25] Tayebati SK, Di Tullio MA, Amenta F (2004). Age-related changes of muscarinic cholinergic receptor subtypes in the striatum of Fisher 344 rats.. Exp Gerontol.

[26] Zagulska-Szymczak S, Filipkowski RK, Kaczmarek L (2001). Kainate-induced genes in the hippocampus: lessons from expression patterns.. Neurochem Int.

[27] Alam AM, Starr MS (1996). Regional changes in brain dopamine utilization during status epilepticus in the rat induced by systemic pilocarpine and intrahippocampal carbachol.. Neuropharmacology.

[28] Smolders I, Sarre S, Vanhaesendonck C, Ebinger G, Michotte Y (1996). Extracellular striatal dopamine and glutamate after decortication and kainate receptor stimulation, as measured by microdialysis.. J Neurochem.

[29] Smolders I, Bogaert L, Ebinger G, Michotte Y (1997). Muscarinic modulation of striatal dopamine, glutamate, and GABA release, as measured with in vivo microdialysis.. J Neurochem.

[30] Wirtshafter D (2004). Role of dopamine D1 receptors in the striatal and cortical fos expression induced by the muscarinic agonist pilocarpine.. Eur J Pharmacol.

[31] Keefe KA, Gerfen CR (1996). D1 dopamine receptor-mediated induction of zif268 and c-fos in the dopamine-depleted striatum: differential regulation and independence from NMDA receptors.. J Comp Neurol.

[32] Konradi C, Leveque JC, Hyman SE (1996). Amphetamine and dopamine-induced immediate early gene expression in striatal neurons depends on postsynaptic NMDA receptors and calcium.. J Neurosci.

[33] Lee B, Cao R, Choi YS, Cho HY, Rhee AD (2009). The CREB/CRE transcriptional pathway: protection against oxidative stress-mediated neuronal cell death.. J Neurochem.

[34] Calabresi P, Centonze D, Gubellini P, Pisani A, Bernardi G (2000). Acetylcholine-mediated modulation of striatal function.. Trends Neurosci.

[35] DeLapp NW, Eckols K, Shannon HE (1996). Muscarinic agonist inhibition of rat striatal adenylate cyclase is enhanced by dopamine stimulation.. Life Sci.

[36] Wang JQ, McGinty JF (1996). Muscarinic receptors regulate striatal neuropeptide gene expression in normal and amphetamine-treated rats.. Neuroscience.

[37] Sperk G, Lassmann H, Baran H, Seitelberger F, Hornykiewicz O (1983). Kainic acid induced seizures: neurochemical and histopathological changes.. Neuroscience.

[38] Lukasiuk K, Dabrowski M, Adach A, Pitkänen A (2006). Epileptogenesis-related genes revisited.. Prog Brain Res.

[39] Cacheaux LP, Ivens S, David Y, Lakhter AJ, Bar-Klein G (2009). Transcriptome profiling reveals TGF-beta signaling involvement in epileptogenesis.. J Neurosci.

[40] Morris TA, Jafari N, DeLorenzo RJ (2000). Chronic DeltaFosB expression and increased AP-1 transcription factor binding are associated with the long term plasticity changes in epilepsy.. Brain Res Mol Brain Res.

[41] Zivin M, Sprah L, Sket D (1996). The D1 receptor-mediated effects of the ergoline derivative LEK-8829 in rats withunilateral 6-hydroxydopamine lesions.. Br J Pharmacol.

